# Synthesis, Properties, and Enzymatic Hydrolysis of Poly(lactic acid)-*co*-Poly(propylene adipate) Block Copolymers Prepared by Reactive Extrusion

**DOI:** 10.3390/polym13234121

**Published:** 2021-11-26

**Authors:** Zoi Terzopoulou, Alexandra Zamboulis, Dimitrios N. Bikiaris, Miguel Angel Valera, Ana Mangas

**Affiliations:** 1 Laboratory of Chemistry and Technology of Polymers and Dyes, Department of Chemistry, Aristotle University of Thessaloniki, GR-541 24 Thessaloniki, Greece; azamboulis@gmail.com (A.Z.); dbic@chem.auth.gr (D.N.B.); 2 AIMPLAS, Asociación de Investigación de Materiales Plásticos Y Conexas, Carrer de Gustave Eiffel, 4, 46980 Valencia, Spain; mavalera@aimplas.es (M.A.V.); amangas@aimplas.es (A.M.)

**Keywords:** poly(lactic acid), poly(propylene adipate), copolymers, biodegradable polymers, ring opening polymerization, reactive extrusion

## Abstract

Poly(lactic acid) (PLA) is a biobased polyester with ever-growing applications in the fields of packaging and medicine. Despite its popularity, it suffers from inherent brittleness, a very slow degradation rate and a high production cost. To tune the properties of PLA, block copolymers with poly(propylene adipate) (PPAd) prepolymer were prepared by polymerizing L-lactide and PPAd oligomers via reactive extrusion (REX) in a torque rheometer. The effect of reaction temperature and composition on the molecular weight, chemical structure, and physicochemical properties of the copolymers was studied. The introduction of PPAd successfully increased the elongation and the biodegradation rate of PLA. REX is an efficient and economical alternative method for the fast and continuous synthesis of PLA-based copolymers with tunable properties.

## 1. Introduction

The negative effects of conventional petrochemical-based plastics on the environment, namely the accumulation of waste [1], marine litter, microplastics [2] and contribution to CO_2_ emissions, have raised concerns about the future of the planet. The most popular approach to minimize the problem, which is recycling, is only performed in less than 10% of the plastic waste (mainly mechanical recycling of poly(ethylene terephthalate)); the majority of it still ends up in landfills [3]. In addition, the COVID-19 pandemic had a profound effect on the increase of municipal and medical waste and disrupted waste management [4]. However, because of the unique characteristics of plastics and their integral role in modern society, their production, as well as waste accumulation, are expected to keep increasing [1].

Plastics are considered one of the world’s most important environmental problems, which led to EU regulations that are designed to help transition to a sustainable plastics economy [5]. To reduce the negative impacts of plastics, various initiatives have been undertaken; the ban of single-use plastics, the regulation of plastic shopping bags, the promotion of the reduce-reuse-recycle approach and raising consumer awareness being the most prevalent ones. To fully transition to a sustainable future, the radical transformation of the plastics market towards biobased and biodegradable polymers is necessary [6]. Many biobased and/or biodegradable plastics with a wide variety of properties have been reported on a research level [7,8], and some are being produced on a commercial scale [9,10]. Among them, poly(lactic acid) (PLA), a biobased, biocompatible, and compostable aliphatic polyester is one of the most produced and used biobased plastics in the world [11]. It is predominantly synthesized via the ring opening polymerization (ROP) of lactide, using organometallic catalysts [12]. PLA is processable with the typical extrusion, injection and blow moulding methods, and its properties can be tuned by changing the L- and D-lactic acid stereoisomer ratio that in turn tunes its crystallinity. Currently, it is used mainly as a packaging and biomedical material [6], and it is the most popular thermoplastic for fused filament fabrication (3D printing) filaments, a newer application that skyrocketed the demand for PLA [13]. The main limitations of PLA that restrict its applications are its inherent brittleness (<10% elongation) and its very slow degradation rate. Even though, for many years, and still to this day, PLA is referred to as biodegradable, it is practically only susceptible to industrial composting [14].

Some of the approaches to overcome the brittleness and slow degradation rate of PLA are copolymerization, blending with other polymers, and addition of plasticizers or fillers [15,16,17,18,19]. The most popular approach to tune the properties of PLA is the copolymerization of L- and D-isomers, and most commercially available PLA grades for packaging applications contain a small amount of the D-isomer [20]. Biomedical grade PLA is often copolymerized with glycolide, ethylene glycol, or monomethyl ether ethylene glycol towards random or block copolymers, respectively [21,22]. These comonomers are used mostly to tune the biodegradation rate and form amphiphilic copolymers. Other lactones have been used, such as ε-caprolactone [23]. When aliphatic polyester pre-polymers are used instead of their monomers in the ROP of PLA, they act as macroinitiators and block copolymers are obtained [24,25,26]. In contrast with PLA polymer blends, block copolymers can be partially miscible or weakly segregated, allowing the second component to act as a plasticizer [27]. In that case, the addition of compatibilizers such as chain extenders or coupling agents is not required. 

Adipic acid (AdA) is considered the most important dicarboxylic acid by the International Energy Agency, because of its use in the synthesis of polyurethanes, polyamides, and its applications as a plasticizer, as well as in food additives and in the cosmetics industry. The production of biobased adipic acid is under development, and the currently known processes are both chemo-enzymatic (with glucose as precursor) and fermentation [28,29,30]. Aliphatic polyesters based on adipic acid and their copolymers are commercially available. The well-known biodegradable poly(butylene-adipate-*co*-terephthalate) (PBAT) finds applications in packaging and as a mulch film [31]. Poly(propylene adipate) (PPAd) was first synthesized in 2007 by the group of Bikiaris, and it had elongation at break ~400% and tensile strength ~11.5 MPa, with low glass transition, melting and cold crystallization temperatures (T_g_ = −57 °C, T_m_ = 29 °C, T_cc_ = −8 °C) [32]. It also crystallizes quite fast due to the large number of methylene groups on its macromolecular chain [33,34].

To incorporate fillers, additives or comonomers into PLA, reactive extrusion (REX) is commonly employed on an industrial scale [12]. Besides compounding, REX can be used to synthesize PLA from lactide in a continuous manner, using a twin-screw extruder [35,36,37,38,39,40]. This method is economically viable, requires very short reaction times (5−10 min), and is a continuous single-stage process [35,41]. The copolymers obtained by REX are very similar to those prepared with batch-type processes, but require a lot less time. 

In this work, poly(propylene adipate) (PPAd) pre-polymer was synthesized with the two-stage melt polycondensation of AdA and 1,3-propanediol (1,3-PD) and used in the synthesis of PLA-*co*-PPAd copolymers for the first time via REX in a torque rheometer as a preliminary step for the scale-up of the process to continuous REX in a twin-screw extruder. The mass of PPAd in the formulation varied from 5 to 25 wt%. Neat PLA from L-lactide (with 5% D-isomer) was also synthesized as a control. Two different reactions temperatures, namely 180 °C and 190 °C were tested. The resulting polymers were characterized by means of their physicochemical properties, including molecular weight determination, evaluation of thermal transition temperatures, tensile properties, hydrophilicity, and hydrolysis rate measurements. 

## 2. Materials and Methods

### 2.1. Materials

Adipic acid (AdA) (purity >99.5%) and 1,3-propanediol (1,3-PD) (purity > 99.6%) were purchased from Fluka (Steinheim, Germany). Titanium butoxide (Ti(OBu)_4_, purity: >97.0%), was purchased from Sigma Aldrich Chemical Co (Steinheim, Germany). L-Lactide (LA) Purlact B3 (purity 99% w/w, stereochemical purity in L-isomer 95% (w/w) was purchased from Corbion N.V. (Gorinchem, The Netherlands) and Tin(II) 2-ethylhexanoate (Sn(Oct)_2_, purity > 92.5%) from Merck KGaA, (Darmstadt, Germany).

### 2.2. Synthesis of Aliphatic Polyesters

#### 2.2.1. Synthesis of Poly(propylene adipate) (PPAd) 

The classic two-stage melt polycondensation method was used for the synthesis of PPAd aliphatic polyester [42,43,44]. In the first stage (esterification), AdA and 1,3-PD in a 1/1.1 molar ratio were charged into the reaction tube in the presence of 400 ppm of Ti(OBu)_4_. The reaction mixture was heated at 180 °C, under N_2_ atmosphere and continuous stirring. The reaction lasted until the theoretical amount of H_2_O produced during the reaction was collected (3.5 h). In the second stage (polycondensation), vacuum (5.0 Pa) was slowly applied to minimize the creation of foam and to avoid PPAd oligomer sublimation, and the polycondensation procedure was carried out at 230 °C for 15 min. $\bar{M_{n}}$ = 6300 g/mol (GPC).

#### 2.2.2. Synthesis of Poly(l-lactide)-*co*-poly(propylene adipate) Copolymers

PLA and its copolymers with PPAd were prepared by REX in a Brabender^®^ Plasti-Corder^®^ Lab-Station torque rheometer. The ROP of lactide was catalysed by Sn(Oct)_2_ in a lactide/Sn(Oct)_2_ molar ratio 1000/1. Triphenyl phosphine (TPP) was used as *co*-catalyst, in equimolar amount to Sn(Oct)_2_ [38]. The catalyst system was added as a solution in dry toluene. PPAd (diol) was added as initiator for the ROP of lactide to obtain PLA-*co*-PPAd copolymers. Blank samples without an initiator (ROP initiated by traces of H_2_O) were also prepared. The polymerization was carried out at temperatures of 180 °C and 190 °C in the mixing chamber, with a 100 rpm screw speed. A small stream of dry N_2_ was circulated to minimize the presence of air in the mixing chamber. The premix of lactide/PPAd/catalyst solution was fed to the rheometer at t = 0. Motor torque (α viscosity) and temperature were monitored vs. time during the polymerization. The samples prepared are listed in Table 1; where 180 and 190 corresponds to the reaction temperature and the ratios 95/5, 85/15 and 75/25 to the feed weight ratios of lactide and PPAd, respectively.

#### 2.2.3. Characterization

The molecular weight of the materials was determined using gel permeation chromatography (GPC) with a Waters 600 high-performance liquid chromatographic pump, Waters Ultrastyragel columns HR-1, HR-2, HR-4E, HR-4 and HR-5, and a Shimadzu RID-10A refractive index detector. For the calibration, 9 polystyrene (PS) standards of molecular weight between 2.5 and 900 kg/mol were employed. The prepared solutions had a concentration of 15 mg /mL, the injection volume was 100 μL and the total elution time was 50 min. The oven temperature was 40 °C. 

Nuclear magnetic resonance (NMR) spectra were recorded in deuterated chloroform for the structural study of monomer and polymer, respectively. An Agilent 500 spectrometer was utilized (Agilent Technologies, Santa Clara, CA, USA), at room temperature. Spectra were calibrated using the residual solvent peaks. 

The morphology of cryofractured cross sections of the samples was studied with a JEOL (Tokyo, Japan) JSM 7610F field emission scanning electron microscope (SEM) operating at 5 kV. 

Differential scanning calorimetry (DSC) analysis was performed using a PerkinElmer Pyris Diamond DSC differential scanning calorimeter (Solingen, Germany) calibrated with pure indium and zinc standards. The system included a PerkinElmer Intracooler 2 (Solingen, Germany) cooling accessory. Samples of 5 ± 0.1 mg sealed in aluminium pans were used to test the thermal behavior of the polymers. Crystallinity degree (X_c_) was calculated with equation X:

(1) $$Xc_{\;}(\%)=\left(\frac{\Delta H_{m}-\Delta H_{\mathrm{cc}}}{\Delta H_{f}^{0}-\frac{1-\mathrm{wt}\%\;\mathrm{PPAd}}{100}}\right)\times 100$$

where Δ*H*_m_, $\Delta H_{\mathrm{cc}}$, $\Delta H_{f}^{0}$ are the experimental melting enthalpy, cold-crystallization enthalpy and the theoretical heat of fusion of 100% crystalline PLA ($\Delta H_{f}^{0}=$93 J/g), respectively.

X-ray Diffraction (XRD) measurements of the polymers and copolymers were performed over the 2*θ* range of 5 to 60°, with steps of 0.05°, scanning speed 1.5 deg/min, using a MiniFlex II XRD system from Rigaku Co. (Tokyo, Japan) with Cu Ka radiation (λ = 0.154 nm).

Infrared (IR) spectra were recorded with a Cary 670 (Santa Clara, CA, USA), Agilent Technologies, equipped with a diamond attenuated total reflectance accessory, ATR, model GladiATR, Pike Technologies. The spectra were recorded in the range 4000–400 cm^−1^ with 32 scans and resolution 4 cm^−1^.

Tensile tests were performed using an Instron 3344 dynamometer (Norwood, MA, USA), in accordance with ASTM D638 using a crosshead speed of 5 mm/min. Dumb-bell-shaped tensile test specimens (central portions 5 mm × 0.5 mm thick, 22 mm gauge length) were prepared by compression moulding in a thermopress at 180 °C, cooled rapidly and cut in a Wallace cutting press. At least five measurements were conducted for each sample, and the results were averaged to obtain the mean values of Young’s modulus, tensile strength at yield and breakpoint, and elongation at break. A student’s T-test was used to determine the statistical significance. 

Water contact angle was measured with an Ossila contact angle goniometer L2004A1 at room temperature (25 °C). Contact angle was measured by gently placing a water droplet (5 µL) on the surface of films of the samples prepared by compression moulding. At least three measurements were performed, and the mean value is reported. A one-way ANOVA test with post hoc Bonferroni correction was performed to determine the effect of the presence of PPAd on the water contact angle of PLA. The software used was IBM SPSS Statistics (IBM, 27). A *p*-value ≤ 0.05 was considered statistically significant.

For the enzymatic degradation testing, thin films prepared by compression moulding (thickness 0.8–1 mm) were cut in squares with dimensions ~1.5 cm × 1.5 cm and were placed in test tubes containing 10 mL phosphate buffer solution (pH 7.4) with 0.01 mg/mL lipase from *Pseudomonas cepacia* and 0.1 mg/mL lipase from *Rhizopus oryzae*. The flasks were then incubated at 36.6 ± 1 °C in an oven for several days while the media were replaced every 3 days. After a specific period of incubation (every 5 days), the samples were removed from the flasks, washed with distilled water, dried under vacuum, and weighed until they remained at a constant weight. The degree of enzymatic hydrolysis was estimated from the mass loss of the samples. Blank incubations were also carried out with the samples in the same buffer without enzyme addition. The degree of biodegradation was estimated by the sample weight loss, according to the following equation:

(2) $$Mass\;loss\;\%=\frac{W_{0}-W_{i}}{W_{o}}$$

Each sample was hydrolyzed in triplicate and the mean value was calculated.

## 3. Results and Discussion

### 3.1. Synthesis of PLA-*co*-PPAd Copolymers

**Figure 1:**
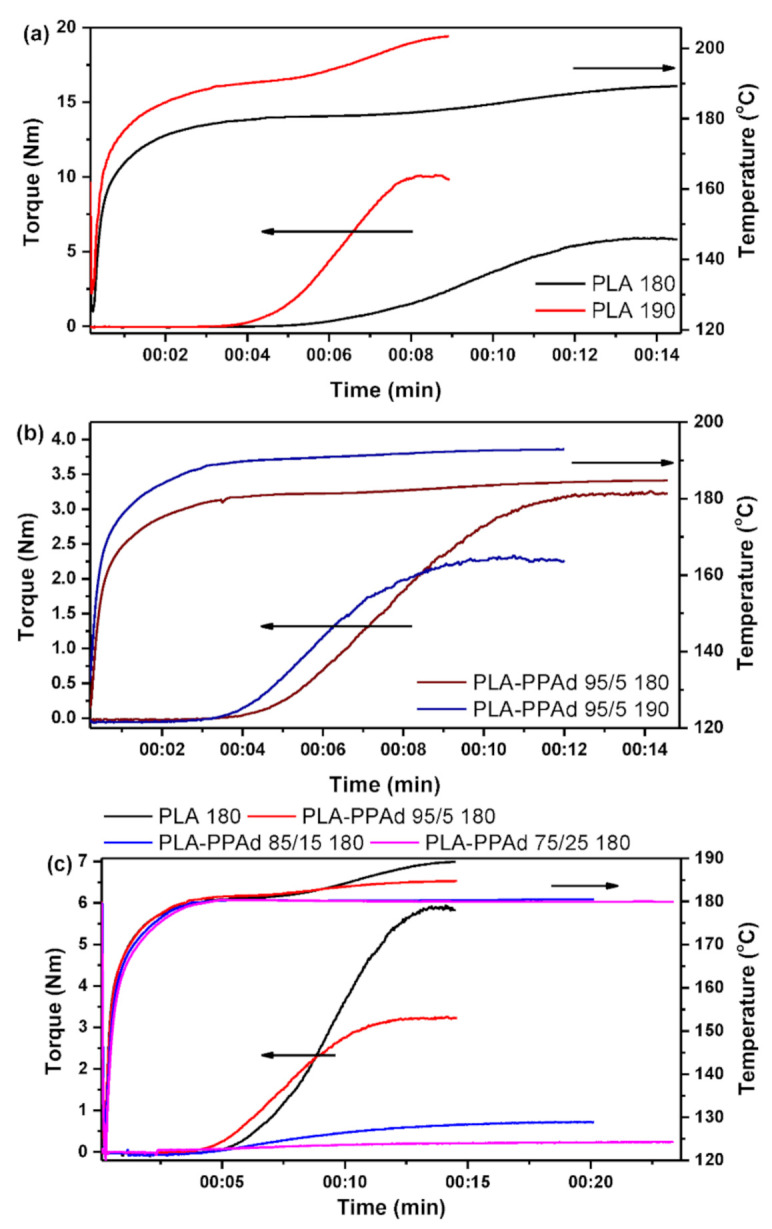
Torque/temperature curves for (**a**) ROP of L-lactide at 180 °C and 190 °C, (**b**) ROP of PLA initiated by PPAd (5 wt%) at 180 °C and 190°C, (**c**) ROP of PLA initiated by PPAd at 5 wt%, 15 wt% and 25 wt% at 180 °C.

The polymerization of PLA was carried out initiated by H_2_O traces present in raw lactide. The rheograms obtained at 180 °C and 190 °C are shown in Figure 1a.

At 190 °C, the ROP of lactide was faster in comparison to 180 °C, reaching the maximum torque at 8.5 min and 14 min, respectively. In addition, the torque (which is proportional to molecular weight or viscosity) was also higher at higher temperature (10.1 Nm at 190 °C vs. 5.9 Nm at 180 °C). However, depolymerization started right after the torque peak was reached, when the mixing chamber temperature was fixed at 190 °C, because the actual melt temperature increased above 200 °C. 

Following the same procedure, PLA-*co*-PPAd copolymers were prepared at 180 °C and 190 °C using 5 wt% of PPAd as macroinitiator for the ROP of L-lactide.

For PLA-PPAd 95/5, the polymerization was faster at 190 °C, reaching stable torque after 11 min, whereas at 180 °C torque stability was reached after 14 min (Figure 1b). However, despite the faster polymerization at higher temperature, the maximum torque (α Mw) was achieved at 180 °C (3.3 Nm versus 2.3 Nm respectively). Therefore, despite the faster polymerization, the temperature for the polymerization of the PLA-*co*-PPAd copolymers was fixed at 180 °C. The torque–temperature curves of all the polymers prepared at 180 °C are shown in Figure 1c.

As PPAd acts as initiator of the ROP of lactide, the higher content of initiator results in faster initiation of the polymerization reaction; however, the maximum Torque (viscosity) is lower due to a larger number of initiation points.

It must be highlighted that at 5 wt% of PPAd, the time required for torque stabilization is similar to blank PLA (12–14 min), and viscosity and melt strength of the PLA-*co*-PPAd is still good for polymer transformation, for example, for injection moulding applications.

For higher PPAd contents, the required residence time to achieve torque stabilization was >20 min and resulted in PLA-*co*-PPAd copolymers with low viscosity and melt strength. Those copolymers would have poor processability by either extrusion or injection moulding technologies; however, they can find applications as base resins in formulations of hot melt adhesives where high viscosity or molecular weight is not required.

### 3.2. Spectroscopic Characterization

**Figure 2:**
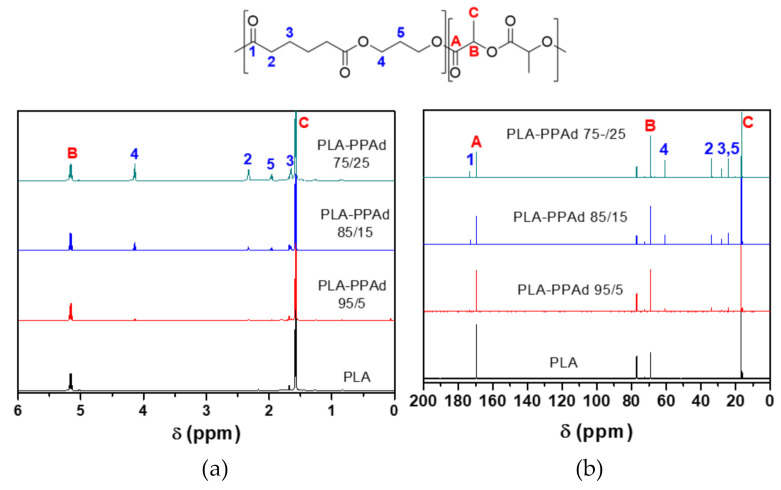
(**a**) ^1^H NMR and (**b**) ^13^C NMR spectra of the samples prepared at 180 °C.

**Figure 3:**
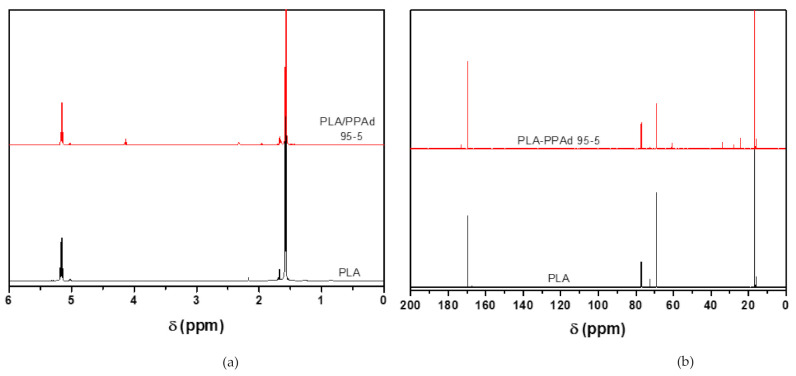
(**a**) ^1^H NMR and (**b**) ^13^C NMR spectra of the samples prepared at 190 °C.

The signals of both PLA (quadruplet peak at 5.16 ppm and double peak at 1.58 ppm) and PPAd (triplet at 4.14 ppm, a broad peak at 2.03 ppm, a multriplet at 1.96 ppm and a multiple peak at 1.66 ppm) are visible in the ^1^H NMR spectra of the PLA-*co*-PPAd copolymers (Figure 2a and Figure 3a). Additionally, traces of unreacted lactide can be observed at 5.03 ppm. In the ^13^C NMR spectra (Figure 2b and Figure 3b), among the other expected resonance signals, the peaks of the two ester carbonyls are clearly visible at 169.5 ppm (PLA) and 173.1 ppm (PPAd), which further confirms successful polymerization. 

The composition of the copolymers was calculated by comparing the integrations of peaks B and 4 of the ^1^H NMR spectra (Figure 2a). It was found to be in complete agreement with the feed ratio (Table 2). Finally, NMR spectra can give some insight about the microstructure of the synthesized copolymers. With a closer look, several small peaks can be observed between 4.1 and 4.4 ppm in PLA-PPAd 85/15 and PLA-PPAd 75/25. One of them was attributed to the CH_2_OC(O) methylene group of a 1,3-PD unit, which is adjacent to a PLA ester, confirming successful copolymerization. Due to their low intensity and some overlapping, the peak could not be integrated to calculate the randomness of the copolymers. Nevertheless, judging from its low intensity, we can safely conclude that the copolymers exhibit a blocky structure with long PLA sequences. 

The ATR spectra of PPAd, PLA, and their copolymers are shown in Figure S1a. PLA shows characteristic IR peaks at 3488 cm^−1^ assigned to O–H bending, at 2994–2944 cm^−1^ of C–H stretching, at 1747 cm^−1^ caused by C=O stretching, at 1452 cm^−1^ of –CH_3_ asymmetric bending, at 1182 cm^−1^ and 1040 cm^−1^ because of the stretching of the –C–O–C– groups [45]. The IR spectra of amorphous and crystalline PPAd have been analyzed in depth in the past [34], and the spectrum recorded here shows the same peaks. While most peaks overlap with those of PLA, two of them witness the presence of the adipic segment; the CH_2_ bending at 1467 cm^−1^ and the C–O/C–C stretch at 1255 cm^−1^. Additionally, the peak of the carbonyl group of PPAd appears at 1720 cm^−1^. The spectra of the copolymers do not show any significant differences from the spectrum of PLA. After closer examination of the carbonyl’s region Figure S1(b), the peak of the copolymers is broader than the PLA homopolymer. A shoulder appears on the peak of the sample PLA-PPAd 85/15 180, and it finally becomes double for 25% PPAd. The emerging of the double carbonyl IR peak is evidence of the presence of PPAd segments in the copolymers. 

### 3.3. Molecular Weight Determination

The molecular weight and polydispersity index (PDI) of all the polymers prepared were measured by GPC. The resulting chromatographs are shown in Figure S2 and the values in Table 3. All chromatographs have only one distribution curve, witnessing the purity of the materials and the successful introduction of the PPAd oligomer in the PLA macromolecular chains. The $\bar{M_{n}}$ of PLA 180 was 75,600 g/mol, which is comparable to commercially available injection moulding grade PLA, while for PLA 190 it was 103,400 g/mol, similar to commercially available PLA appropriate for film production [46]. Similar $\bar{M_{n}}$ values that were reported in the literature [23,36,40]. When prepared in a melt polycondensation apparatus, with reaction times >2 h, PLA-*co*-PPAd copolymers had $\bar{M_{n}}$ = 47,700 and 53,700 g/mol for PPAd content 15 wt% and 31 wt% respectively [47], which are very close to the values obtained by REX, for much smaller reaction times.

While PLA synthesized at 190 °C has higher molecular weight than when synthesized at 180 °C, the first copolymer that was tested (PLA-PPAd 95/5) had a better molecular weight when the ROP took place at 180 °C. In agreement with the motor torque monitoring during reactive extrusion, the molecular weight of the samples decreases after the addition of PPAd, and with increasing the PPAd content. The free hydroxyl groups of the PPAd oligomers act as a *co*-initiator for the ROP, resulting in the observed smaller molecular weight values. 

**Scheme 1:**
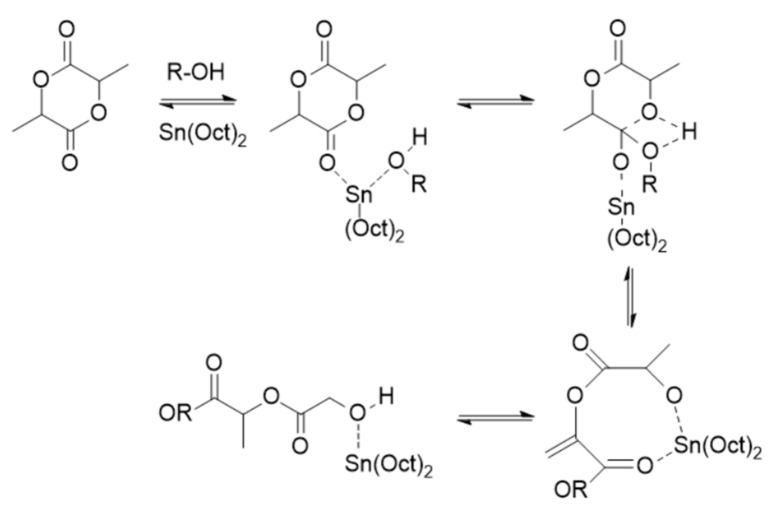
Coordination-insertion mechanism of the ROP of lactide using Tin(II) octoate [50].

ROP is a propagation process of cyclic monomers. The process can be initiated by ions, and the ROP mechanisms can be anionic, cationic, or coordination–insertion [48]. The most common catalyst system utilized for lactide polymerization is Sn(Oct)_2_ with the addition of an initiator. Sn(Oct)_2_ catalyzes the ROP via a coordination-insertion mechanism (Scheme 1) [49]. Traces of water are also enough to initiate the ROP of lactide [49]. While Sn(Oct)_2_ promotes fast polymerization, it also causes undesirable inter- and intramolecular transesterification reactions that ultimately lead to low molecular weight. 

TPP is a Lewis base that is used as an initiator and increases the polymerization rate of lactide and when combined with equimolar amounts of Sn(Oct)_2_; it allows for reaching an acceptable balance between propagation and depolymerization rates, essential for the ROP of lactide in an extruder [37,38]. Additionally, it leads to products with a narrower size distribution and without racemization reactions [38]. This is believed to occur because the coordination of the nucleophilic TPP on the metal atom polarizes the generated metal alkoxide, thus making the insertion of the monomer into the metal alkoxide bond of the initiator easier. 

When adding the PPAd oligomers in the reaction mixture, their hydroxyl and carboxyl end groups are expected to participate in the reaction. By increasing the concentration of hydroxyls, the number of initiation sites is increased, resulting in more inactive PLA chains and fewer active tin alkoxide species, ultimately reducing the molecular weight [37]. Therefore, the larger the quantity of PPAd added, the smaller the molecular weight. While hydroxyls do not significantly affect monomer conversion, but do reduce the molecular weight, carboxyl end groups are expected to decelerate the reaction. PPAd reduced the molecular weight of PLA when the copolymers were prepared by melt polycondensation as well [47].

### 3.4. Scanning Electron Microscopy (SEM)

One method to investigate whether two polymers formed blends is to observe their cryofractured surfaces. In polymer blends, two distinct phases can usually be identified, with phase separation present when the different components are incompatible or immiscible. In the SEM micrographs of Figure S3, no evidence of a second phase exists on the surfaces of the PLA-*co*-PPAd copolymers with PPAd content up to 15 wt%. The surface of PLA-PPAd 75/25 (Figure S3d) shows evidence of a second phase which could be PPAd, in the form of spherical particles, that are fully embedded in the PLA matrix. Lack of phase separation also supports the NMR conclusion that the materials are not blends, but copolymers. 

### 3.5. Thermal Properties and Crystallinity

**Figure 4:**
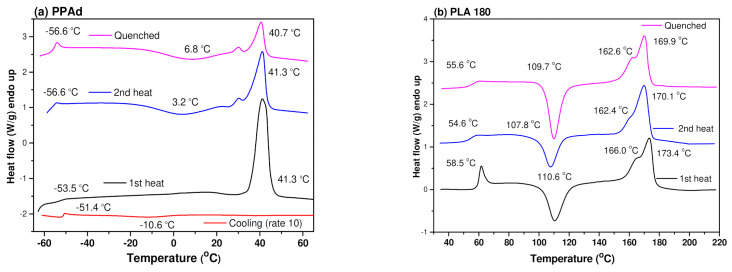

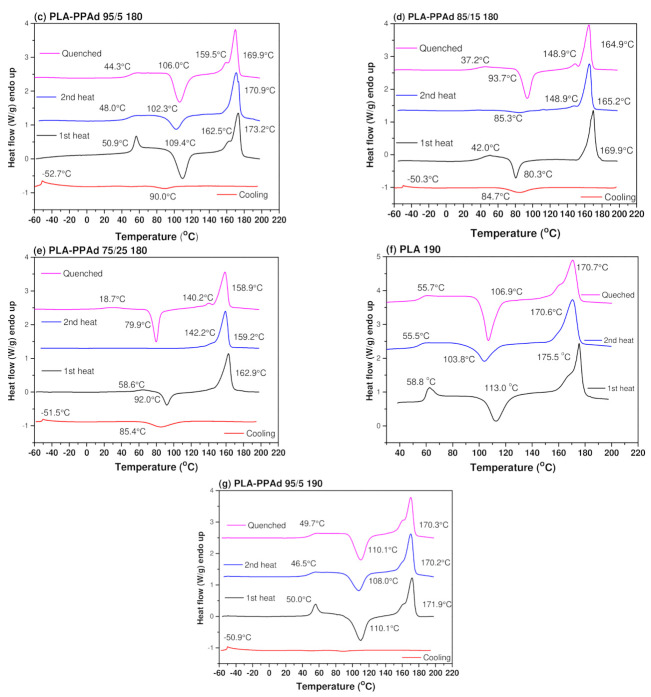
DSC traces of (**a**) PPAd, (**b**) PLA 180, (**c**) PLA 190, (**d**) PLA-PPAd 95/5 180, (**e**) PLA-PPAd 95/5 190, (**f**) PLA-PPAd 85/15 180, (**g**) PLA-PPAd 75/25 180.

The DSC traces of PLA and its copolymers with PPAd are presented in Figure 4. The 1st heat corresponds to the as-received materials, which was followed by cooling with rate 10 °C/min, a 2nd heating step, quenching, and finally re-heating of the quenched sample to obtain the glass transition (T_g_). All heating steps were performed with a rate of 20 °C/min. 

PPAd (Figure 4a) was received semicrystalline, as witnessed by its melting during the 1st heating scan. Its melting point (T_m_) was observed at 41 °C, and its T_g_ after quenching was −57 °C. These values are consistent with previous reports [33,34,47,51,52,53]. The quenched PPAd also showed cold crystallization at T_cc_ = 6.8 °C and melt crystallization at T_c_ = −10.6 °C. PPAd exhibited a multiple melting behavior, since a secondary melting peak is recorded in its reheating scans at about 30 °C, right after the end of cold crystallization. This phenomenon has been observed before for PPAd [33] and is attributed to the melting of less perfect crystallites that did not have enough time to melt and recrystallize. 

The DSC traces of PLA 180 (Figure 4b) show the glass transition, cold crystallization and multiple melting behavior in the common temperature ranges for PLA of medium molecular weight [54,55]. The T_g_ of the melt-quenched PLA was observed at 55.6 °C; it crystallizes during heating with a peak at T_cc_ = 109.7 °C and the formed crystals partially melted at 162.2 °C, and finally at 169.9 °C, where the main melting endotherm is positioned. Multiple melting of PLA is ascribed to crystal reorganization during heating, and it is affected by the crystallization temperature [49]. The as-received PLA had a crystallinity of about 15%, as calculated by Equation (1); however, it did not crystallize significantly during cooling with the applied rate. After the introduction of PPAd, the DSC curves remain similar to those of neat PLA (Figure 4c–e), with all copolymers retaining the semicrystalline character. Significant shifting of the peak temperatures is, however, observed. Neither melting, crystallization peaks nor glass transition that might arise from PPAd moieties exclusively exist. In contrast, blends of PLA with PPAd prepared by solution mixing showed two melting peaks that correspond to each polymer, proving their immiscibility [49]. 

**Figure 5:**
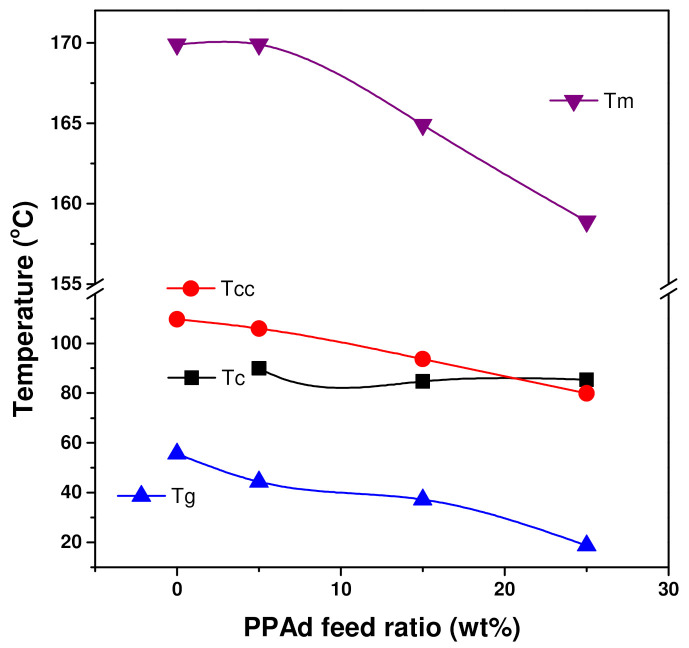
Effect of PPAd content on the thermal transition temperatures of PLA. (The lines are just guides for the eye).

The effect of PPAd content on the characteristic thermal transitions of PLA, deducted from the DSC curves, is presented in Figure 5. T_cc_, T_m_ and T_g_ were obtained from the heating of the melt-quenched samples with a rate of 20 °C/min, and the T_c_ from the cooling run with rate 10 °C/min. T_cc_, T_m_ and T_g_ decreased by increasing the PPAd content. PPAd both reduces the molecular weight and acts as a plasticizer to PLA, which results in the decrease of the T_g_ and T_m_. In contrast with neat PLA, which crystallized insignificantly during cooling with the applied rate, all copolymers showed detectable melt crystallization peaks at around 85–90 °C, which may be a result of the lower molecular weight of the copolymers, as well as the mobility of the aliphatic macromolecular chains of PPAd. 

**Figure 6:**
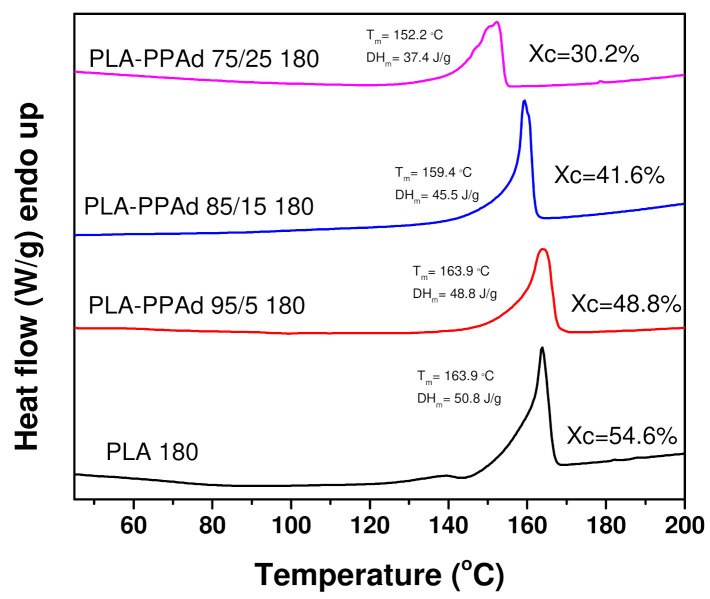
DSC curves of the materials prepared at 180 °C after cold crystallization at 85 °C for 1 h.

DSC curves of the materials prepared at 180 °C were also recorded after cold crystallization at 85 °C for 1 h (Figure 6). Cold crystallization of amorphous PLA pellets is typically performed before their processing, so it does not become sticky or forms clumps during drying [56]. PPAd decreased the final degree of crystallinity of PLA, as calculated by Equation (1), a phenomenon that was also observed in block copolymers PCL and PPAd [57]. Still, all copolymers successfully crystallized in the same temperature as the PLA homopolymer.

**Figure 7:**
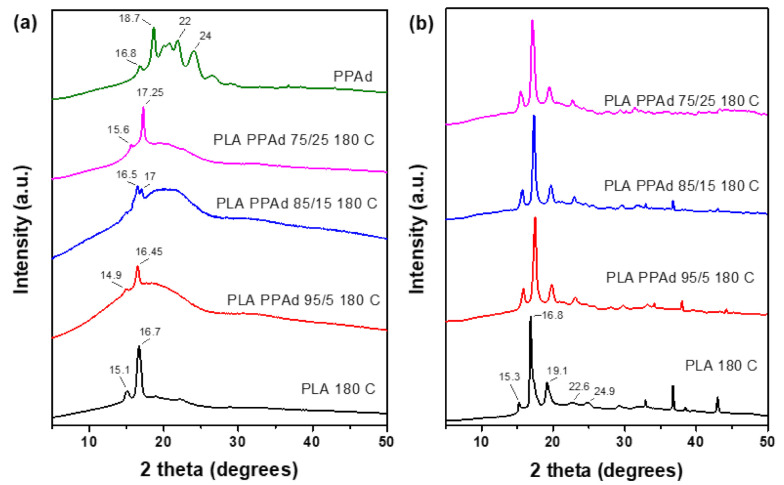
X-Ray diffractograms of PPAd, PLA and their copolymers (**a**) as received from the extruder and (**b**) after cold-crystallization at 85 °C for 1 h.

X-ray diffraction patterns of the materials as-received from the extruder and of PPAd are presented in Figure 7a. PPAd has several crystalline reflections, typical for aliphatic polyesters with an odd number of methylene groups, at 16.8. 18.7, 20.8, 22 and 24 °, which agree with previous reports [33,58,59]. Its crystal structure hasn’t been identified so far. PLA showed two strong reflections at 15.1 and 16.7 °, which correspond to the (010) and (200/110) crystal planes. Its unit cell is orthorhombic [60]. After annealing (Figure 7b), PLA showed an additional strong reflection at 19 ° of the plane (203). The copolymers were received quite amorphous, witnessed by the large halo in the patterns, and after annealing they show the same reflection peaks as PLA. No strong peaks of PPAd can be detected, which implies that crystals of PPAd were not formed, since it crystallized in temperatures lower than room temperature. In a previous study, it was found that PPAd blocks were not able to crystallize in copolymers with PCL [57]. 

### 3.6. Tensile Properties

The PLA homopolymer was very brittle and could not be tested, because the specimens would break during handling. The two samples PLA-PPAd 85/15 and PLA-PPAd 75/25 were the only ones that could be molded into dumbbell shapes specimens for testing; therefore at least 15 wt% of PPAd oligomer must be added in the synthesis of PLA copolymers to yield adequately plasticized materials. The small amounts of unreacted lactide (see Table 2) can also act as plasticizers for PLA. 

**Figure 8:**
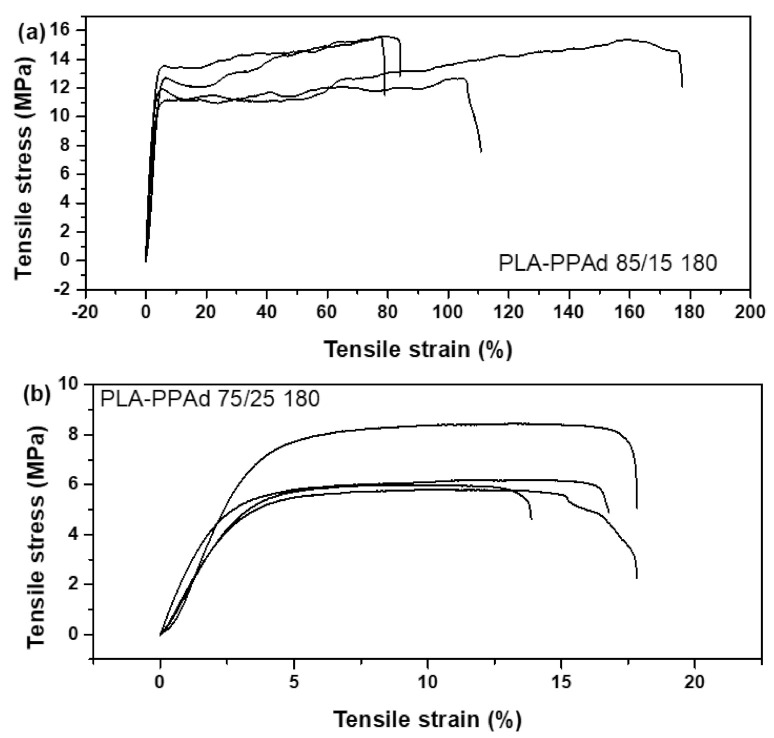
Indicative stress–strain curves of repeated measurements of (**a**) PLA-PPAd 85/15 and (**b**) PLA-PPAd 75/25 (amorphous).

The stress–strain curves are shown in Figure 8 and the resulting parameters are shown in Table 4. The elongation at break of PLA with similar molecular weight as PLA 180 prepared for this work ranges from 1–3% [61]. The typical stress–strain curve of PLA does not have a yield point due to the inherent brittleness of the polymer. With the addition of 15 wt% PPAd, the stress–strain curves (Figure 8a) exhibited a yield point, and significantly larger elongation at break (109.1 ± 45.9%) in comparison with previously reported tensile properties of PLA [45]. The addition of 25 wt% PPAd led to soft and weak materials because of the low molecular weight. 

### 3.7. Water Contact Angle and Enzymatic Hydrolysis

The factors that affect the biodegradability of polymers are their chemical structure, hydrophilicity, molecular weight, melting point and crystallinity [62,63]. PLA has been considered a biodegradable material as per the definition of IUPAC, which states that biodegradale materials are “polymers susceptible to degradation by biological activity, with the degradation accompanied by a lowering of its molar mass” [64]. Biodegradable plastics are recommended by the European Commission for applications where it is not possible to reduce, reuse or recycle [65]. PLA however is not degradable in an open environment, and requires industrial composting conditions. This very slow rate of degradation is caused by the side methyl group that prevents water and enzymes from easily approaching the labile ester bond.

**Figure 9:**
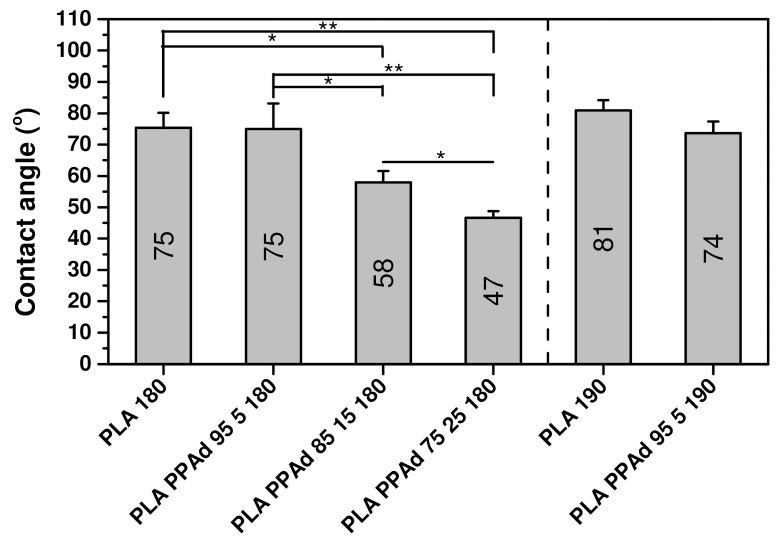
Water contact angle of PLA and the PLA-*co*-PPAd copolymers. * *p* < 0.05, ** *p* < 0.01.

To explore the effect of hydrophilicity on the hydrolysis rates of polymers, their water contact angle was measured. The effect of PPAd content on the water contact angle of PLA is shown in Figure 9. The water contact angle of polymers depends on their chemical composition and surface properties (roughness, heterogeneity, preparation method), as well as temperature [66]. All contact angle values were <90 °, witnessing the high surface energy and overall wettability of all polymers owning the presence of many polar groups on their molecular structure. PLA had a contact angle of 75 ± 4.8 °, in line with previous reports [67]. To significantly increase the hydrophilicity of PLA, at least 15 wt% of PPAd must be added. As the PPAd content increased to 25 wt%, the contact angle was further reduced. The increased hydrophilicity can be primarily attributed to the smaller molecular weights of the copolymers, which mean increased concentrations of hydrophilic -OH and -COOH groups. 

**Figure 10:**
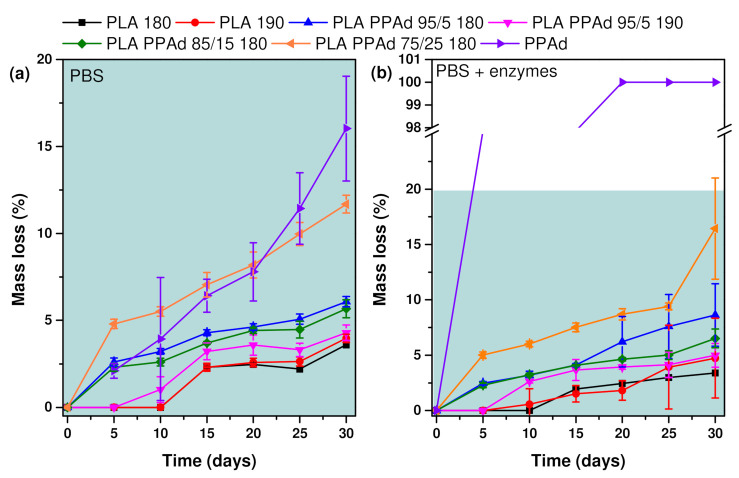
% Mass loss of the samples during incubation at 37 °C in (**a**) PBS and (**b**) PBS with enzymes (0.01 mg/mL lipase from *Pseudomonas cepacia* and 0.1 mg/mL lipase from *Rhizopus oryzae*). The shaded sections correspond to 20% mass loss.

The biodegradation of polymers can be monitored with several different methods, including measuring their weight loss during incubation in the presence of enzymes, and microscopic observation of their surfaces. To diminish the effect of crystallinity and dimensions, the specimens used were amorphous films of the same size and similar thickness. In Figure 10, the mass loss of PLA and its copolymers for 30 days of (a) hydrolysis and (b) enzymatic hydrolysis is shown. Cleavage of ester bonds can be catalyzed by enzymes such as lipases, but it can also take place in their absence, since they are susceptible to hydrolytic attack. The PLA homopolymer lost about 3.5% of its mass after 1 month. The incorporation of PPAd moieties increased the degradation rate, since it is susceptible towards hydrolytic and enzymatic degradation (~15% mass loss without enzymes, 100% mass loss with enzymes). Increasing its content from 5 to 15 wt% did not significantly affect mass loss; however, further increasing it to 25% led to a copolymer much more prone to hydrolysis. These results are in line with the contact angle measurements, suggesting that hydrophilicity is the main parameter that controls the biodegradability of the copolyesters. 

**Figure 11:**
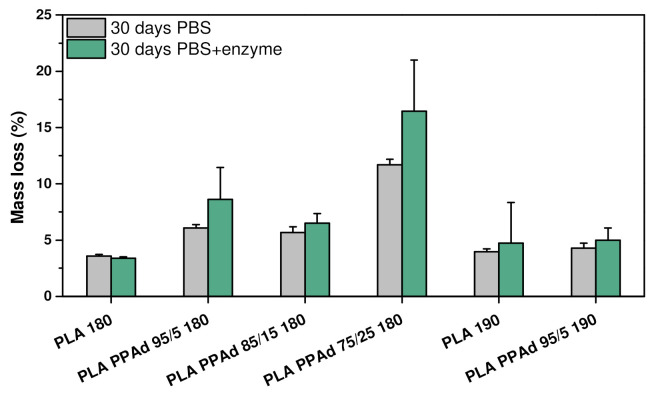
Effect of the presence of enzymes in the hydrolysis of the samples for 30 days.

The effect of the presence of the enzymes of the final mass loss of the polyesters is presented in Figure 11. The mass loss of PLA did not change significantly in the presence of enzymes, revealing its limited susceptibility to the lipases used. PPAd on the other hand completely degraded after 20 days of incubation with the enzymes, while without them it lost about 7.5% of its initial mass. Therefore, PPAd can be efficiently degraded by lipases and as a result, the PLA-*co*-PPAd copolymers lose more weight as they are more prone to be attacked by the enzymes in comparison with neat PLA. Similar results were obtained in previous work on PLA-PPAd blends [51].

**Figure 12:**
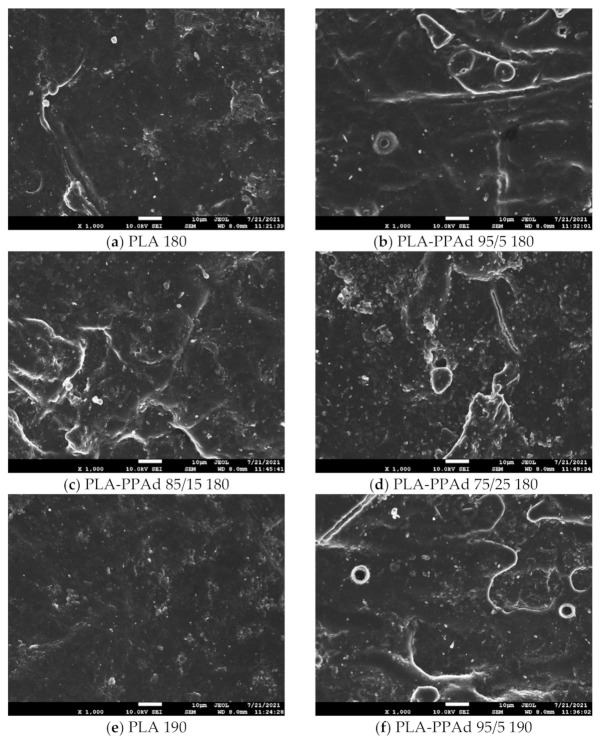
SEM micrographs of the surfaces of the samples after 30 days of enzymatic hydrolysis.

The morphology of the sample’s surfaces after 30 days of enzymatic hydrolysis was studies with SEM (Figure 12). The surface of the PLA homopolymer did not possess many irregularities, while all the copolymers showed significant deterioration of their surfaces, increased roughness, and holes as well as ridges are visible. These observations are in agreement with the mass loss measurements. 

## 4. Conclusions

PLA-*co*-PPAd block copolymers were successfully prepared by REX in a torque rheometer, showing potential to be scaled-up by REX in a twin-screw extruder, in an economic and fast way. The optimum reaction temperature was 180 °C, and the reactions were completed in very short times (up to 20 min) in comparison with the typical batch-type processes. The molecular weight of the materials was comparable to similar copolymers synthesized with typical ROP and melt polycondensation reactions that require several hours to be completed. The PLA moieties are those that were responsible for crystallization. The addition of 15 wt% PPAd in the feed successfully increased the elongation of PLA to ~100%, making the addition of a plasticizer unnecessary. PPAd acted as a macroinitiator that reduced the molecular weight of PLA; however, the produced copolymers can find applications as films or base resins in formulations of hot melt adhesives where high viscosity is not required. Finally, PPAd significantly increased the hydrolysis rates of PLA copolymers, which can be controlled by adjusting the PPAd content. 

## Figures and Tables

**Table 1:** List of the homopolymers and copolymers prepared.

Sample Name
PLA 180
PLA-PPAd 95/5 180
PLA-PPAd 85/15 180
PLA-PPAd 75/25 180
PLA 190
PLA-PPAd 95/5 190

**Table 2:** Unreacted lactide and comonomer molar ratios calculated by NMR.

Temperature	180 °C				190 °C	
Sample	PLA	PLA-PPAd 95/05	PLA-PPAd 85/15	PLA-PPAd 75/25	PLA	PLA-PPAd 95/05
Unreacted lactide (%)	5.8	5.8	7.1	6.7	5.6	5.8
PLA-PPAd ratio	-	94/06	84/16	74/26	-	94/06

**Table 3:** Molecular weight and PDI (GPC) of PLA and the copolymers with PPAd.

Sample	$\bar{M_{n}}(g/\mathrm{mol})$	$\bar{M_{w}}(g/\mathrm{mol})$	$\bar{M_{p}}(g/\mathrm{mol})$	PDI
PLA 180	75,600	119,700	103,500	1.58
PLA-PPAd 95/5 180	63,300	94,100	88,800	1.48
PLA-PPAd 85/15 180	41,200	55,000	54,800	1.33
PLA-PPAd 75/25 180	29,400	39,300	38,500	1.34
PLA 190	103,400	161,300	134,200	1.56
PLA-PPAd 95/5 190	60,100	88,200	80,300	1.46
PPAd	6300	11,340	10,800	1.8

*M*_n_: Number Average Molecular Weight, *M*_w_: Weight Average Molecular Weight, *M*_p_: peak maximum molecular mass, PDI: polydispersity index.

**Table 4:** Tensile test results. Student’s t-test between the two samples yielded *p* < 0.05 for tensile stress, elongation and Young’s modulus.

Sample	Tensile Stress at Break	Stress at Yield	Elongation	Young’s Modulus
	MPa	MPa	%	MPa
PLA-PPAd 85/15	14.8 ± 1.4	12.3 ± 1	109.1 ± 45.9	391.8 ± 50.1
PLA-PPAd 75/25	7.3 ± 1.1	-	15.4 ± 1.8	218.3 ± 28.1

## Data Availability

Data is contained within the article or supplementary material.

## Supplementary Materials

The following are available online at https://www.mdpi.com/article/10.3390/polym13234121/s1, Figure S1. GPC chromatographs. Figure S2. (a) ATR spectra of PPAd, PLA and their copolymers, (b) zoom in the 1800–1650 cm-1 region of PPAd and the materials prepared at 180 °C. Figure S3. SEM micrographs of cryofractured surfaces in different magnifications (×1500 and ×3500).
