# Supplementary material for: Synthesis, Properties, and Enzymatic Hydrolysis of Poly(lactic acid)-co-Poly(propylene adipate) Block Copolymers Prepared by Reactive Extrusion
Source: Polymers (Basel). 2021 Nov 26;13(23):4121. doi: 10.3390/polym13234121 (PMC8659515; doi:10.3390/polym13234121)
Supplement: Supplementary file 1 [file polymers-13-04121-s001.zip › polymers-1469214-supplementary.pdf]

Supplementary Materials

Synthesis, Properties, and Enzymatic Hydrolysis of Poly(lactic acid)-co-poly(propylene adipate) Block Copolymers Prepared by Reactive Extrusion

Zoi Terzopoulou [1,*], Alexandra Zamboulis [1], Dimitrios N. Bikiaris [1], Miguel Angel Valera [2] and Ana Mangas [2]

[1] Laboratory of Chemistry and Technology of Polymers and Dyes, Department of Chemistry, Aristotle University of Thessaloniki, GR-541 24 Thessaloniki, Greece; azamboulis@gmail.com (A.Z.); dbic@chem.auth.gr (D.B.)

[2] AIMPLAS, Asociación de Investigación de Materiales Plásticos Y Conexas, Carrer de Gustave Eiffel, 4, 46980 Paterna, Valencia, Spain ; mavalera@aimplas.es (M.A.V.); amangas@aimplas.es (A.M.)

* Correspondence: terzozoi@chem.auth.gr

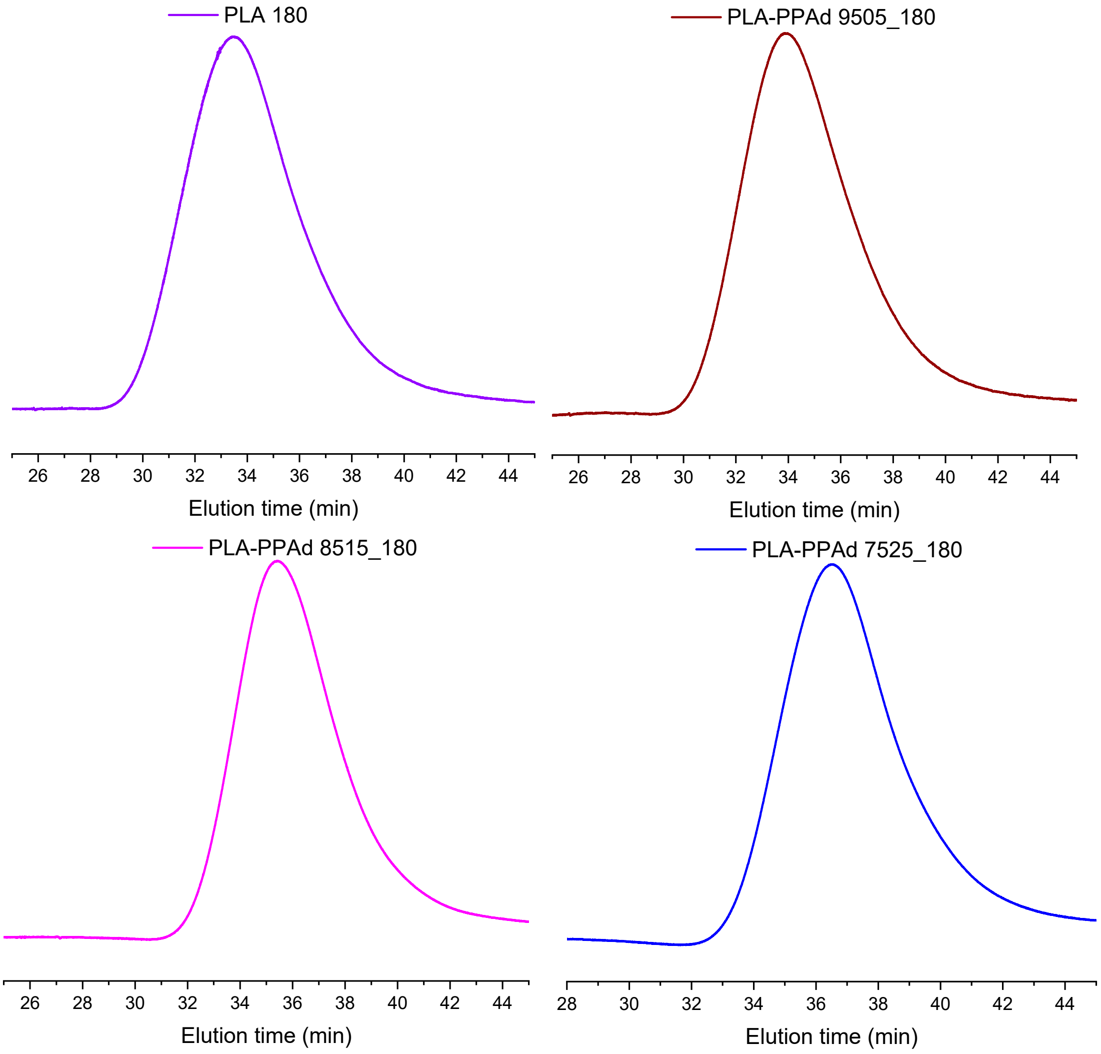

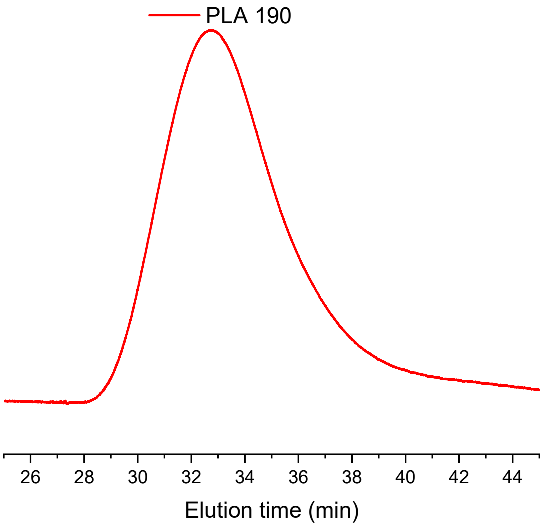

Figure S1. GPC chromatographs.

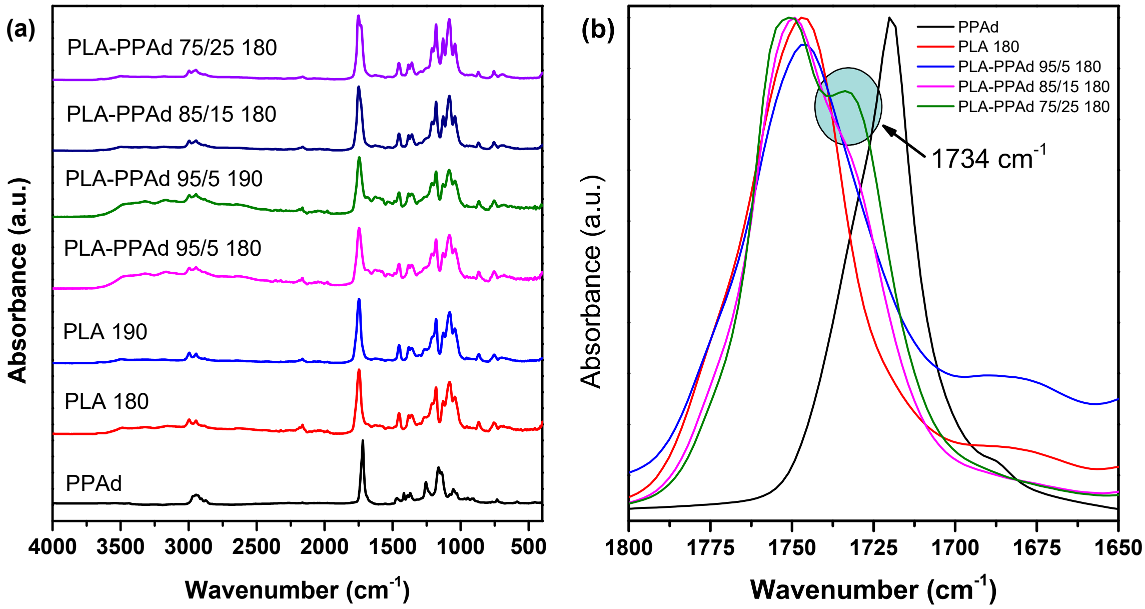

Figure S2. (a) ATR spectra of PPAd, PLA and their copolymers, (b) zoom in the 1800-1650 cm^{-1} region of PPAd and the materials prepared at 180 °C.

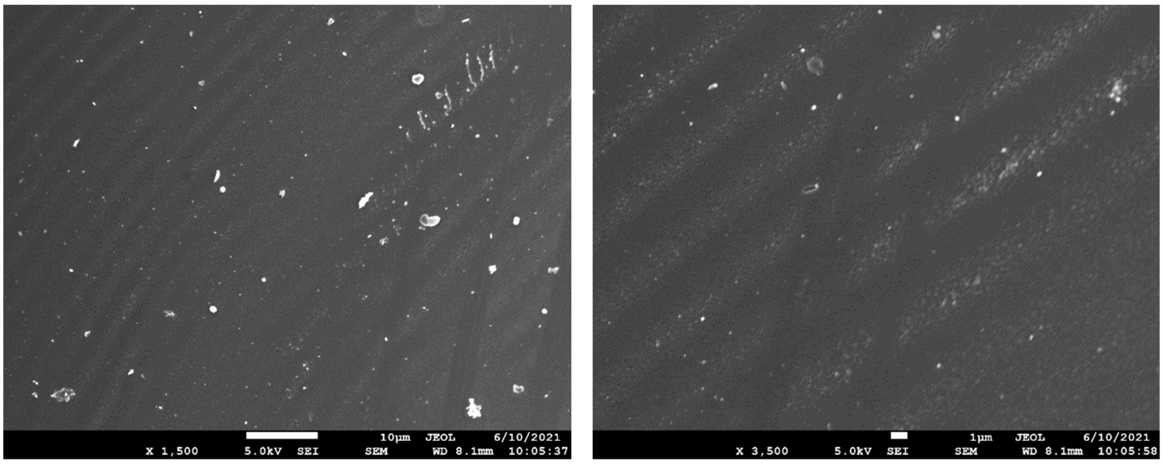

(a) PLA 180

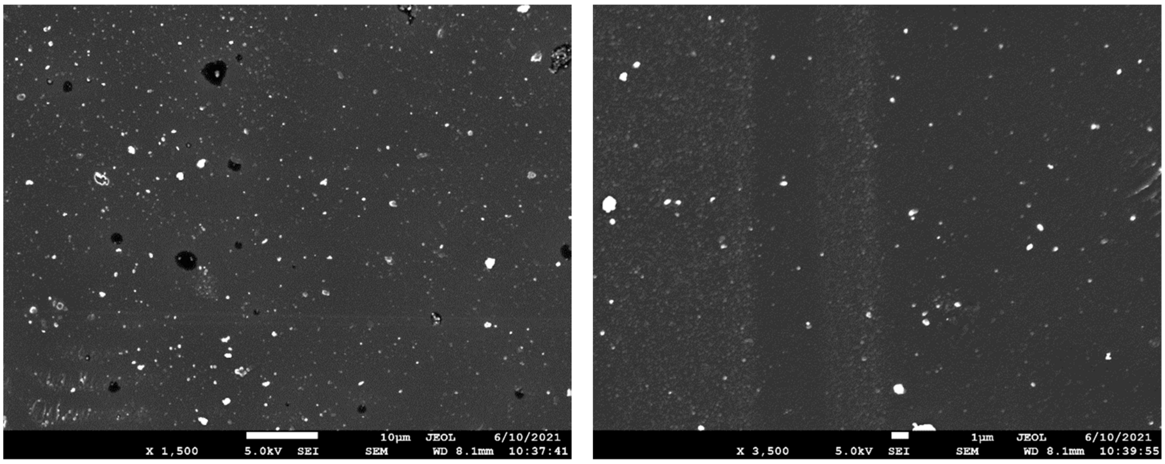

(b) PLA-PPAd 95/5 180

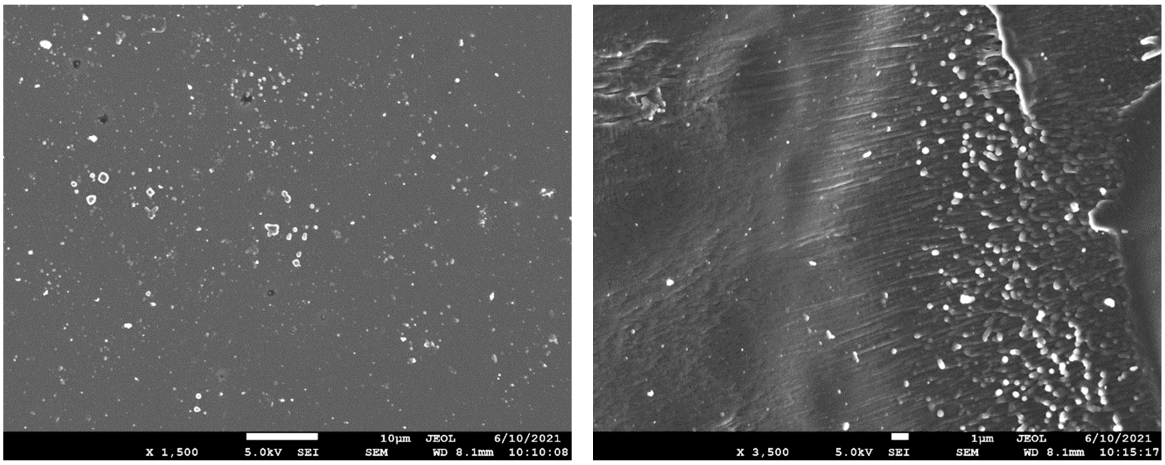

(c) PLA-PPAd 85/15 180

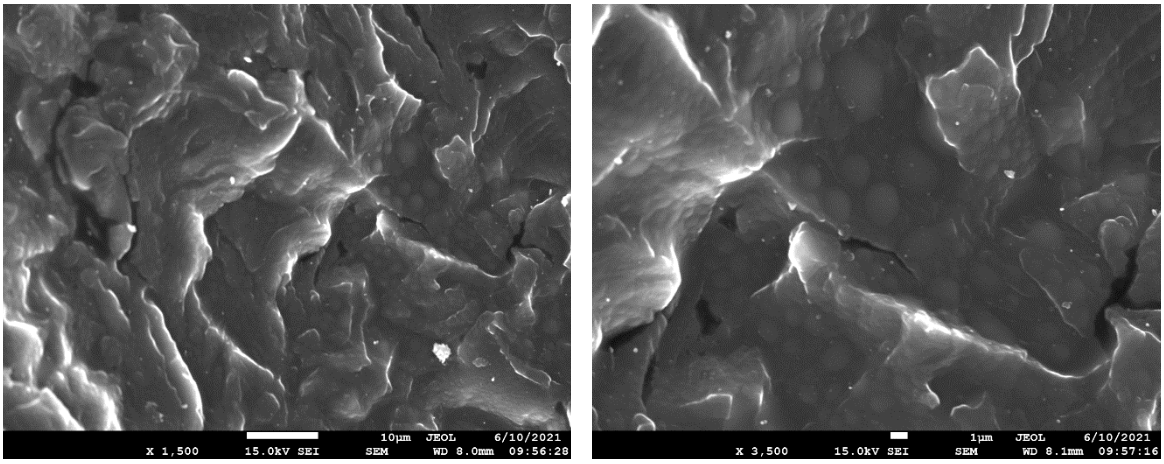

(d) PLA-PPAd 75/25 180

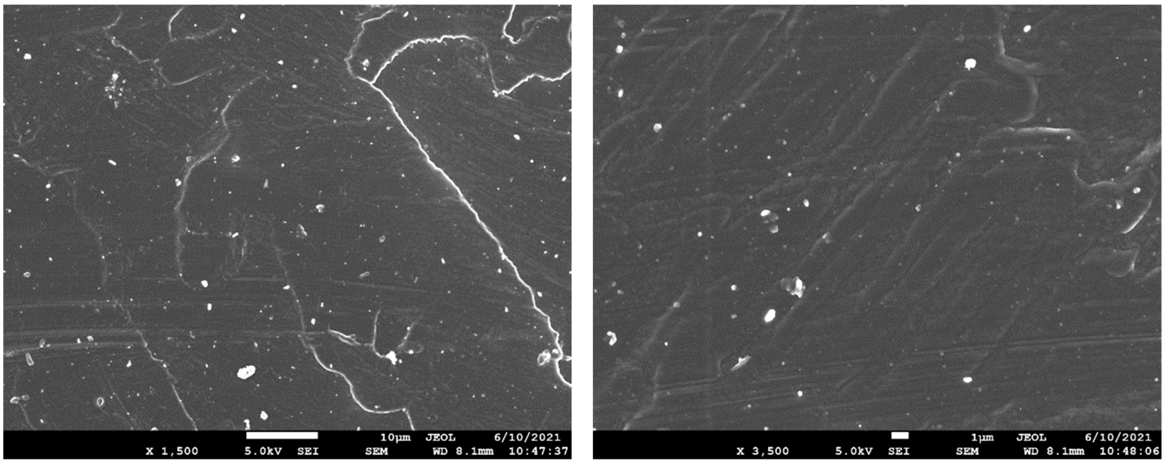

(e) PLA 190

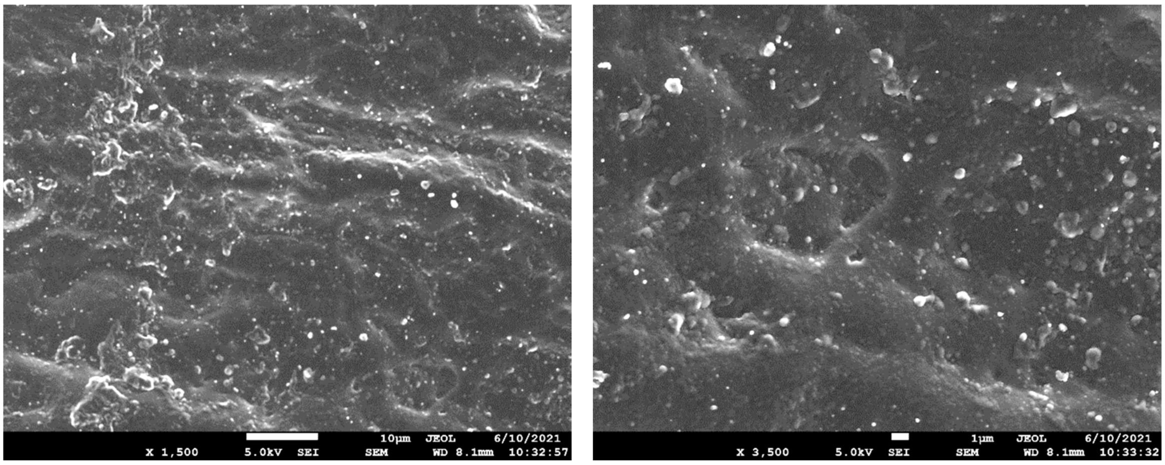

(f) PLA-PPAd 95/5 190

Figure S3. SEM micrographs of cryofractured surfaces in different magnifications (x1500 and x3500).
